# Making Better Influenza Virus Vaccines?

**DOI:** 10.3201/eid1201.051043

**Published:** 2006-01

**Authors:** Peter Palese

**Affiliations:** * Mount Sinai School of Medicine, New York, New York, USA

**Keywords:** Influenza virus, Live vaccine, Killed vaccine, Reverse genetics, Cold-adapted vaccine. Universal vaccine, Adjuvant, Interferon, NS1 vaccine, Recombinant virus, Perspective

## Abstract

Killed and live influenza virus vaccines are effective in preventing and curbing the spread of disease, but new technologies such as reverse genetics could be used to improve them and to shorten the lengthy process of preparing vaccine seed viruses. By taking advantage of these new technologies, we could develop live vaccines that would be safe, cross-protective against variant strains, and require less virus per dose than conventional vaccines. Furthermore, pandemic vaccines against highly virulent strains such as the H5N1 virus can only be generated by reverse genetics techniques. Other technologic breakthroughs should result in effective adjuvants for use with killed and live vaccines, increasing the number of available doses. Finally, universal influenza virus vaccines seem to be within reach. These new strategies will be successful if they are supported by regulatory agencies and if a robust market for influenza virus vaccines against interpandemic and pandemic threats is made and sustained.

## Abstract 2

Better influenza vaccines are possible and necessary.

Influenza virus vaccines were first developed in the 1940s and consisted of partially purified preparations of influenza viruses grown in embryonated eggs. Because of substantial contamination by egg-derived components, these killed (formaldehyde-treated) vaccines were highly pyrogenic and lacking in efficacy. A major breakthrough came with the development of the zonal ultracentrifuge in the 1960s (invented by Norman G. Anderson) (*1*). This technology, which originated from uses for military purposes, revolutionized the purification process and industrial production of many viruses for vaccines. To this day, it remains the basis for the manufacturing process of our influenza virus vaccines.

Current influenza virus vaccines consist of 3 components: an H1N1 (hemagglutinin [HA] subtype 1; neuraminidase [NA] subtype 1), an H3N2 influenza A virus, and an influenza B virus. Specifically, the 2005–2006 vaccine formulation is made up of the A/New Caledonia/20/99 (H1N1), A/California/7/2004 (H3N2), and B/Shanghai/361/2002 viruses. Changes in the HA of circulating viruses (antigenic drift) require periodic replacement of the vaccine strains during interpandemic periods. The World Health Organization publishes semiannual recommendations for the strains to be included for the Northern and Southern Hemispheres (*2*). To allow sufficient time for manufacture, in the United States the US Food and Drug Administration (FDA) determines in February which vaccine strains should be included in the following winter's vaccine. Unfortunately, FDA recommendations are not always optimal. For example, in 2003 FDA rejected the use of the most appropriate H3N2 strain, A/Fujian/411/2002, and instead again used the same strain as in the 2002 formulation. This decision was made primarily because the A/Fujian/411/2002 strain had first been isolated in Madin Darby canine kidney (MDCK) cells rather than in embryonated eggs. Use of MDCK cells for virus isolation is not allowed by FDA's rules, which do not yet encompass advanced technologies or scientifically sound purification procedures based on limiting dilutions or cloning with DNA. Because of this bureaucratic roadblock, the H3N2 component of the 2003–2004 influenza virus vaccine was antigenically "off" and showed suboptimal efficacy. One hundred fifty-three pediatric deaths were associated with influenza infections during the 2003–2004 season in 40 states, whereas only 9 such deaths had been reported in the following season (*3*). Also, because the cumbersome classical reassortment technique used for preparing the appropriate seed strains makes the yearly process of manufacturing influenza virus vaccines unnecessarily lengthy, new variants first appearing early in the season are rarely considered for the vaccine formulation of the following winter.

## Currently Licensed Influenza Virus Vaccines

Most influenza virus vaccines used in the United States and Europe consist of embryonated egg-grown and formaldehyde-inactivated preparations, which, after purification, are chemically disrupted with a nonionic detergent (for example, Triton X-100). The split virus preparations show lower pyrogenicity than whole virus vaccines. In general, 1 dose for adults contains the equivalent of 45 μg HA (15 μg HA for each of the 3 antigenic components). This dose is approximately the amount of purified virus obtained from the allantoic fluid of 1 infected embryonated egg. If 100 million doses of killed influenza virus vaccine are prepared, the manufacturer has to procure 100 million embryonated eggs. Clearly, this manufacturing process is dependent on the timely availability of embryonated eggs and the vaccine seed strains to be used in a particular season. Most of these prototype seed strains are provided to the manufacturers by government agencies, which create high-yielding strains through classical reassortment with a high-yielding laboratory strain, A/PR/8/34, following the procedures designed by Kilbourne (*4*). Unfortunately, only (high-yielding) influenza A viruses can be made in this way, and even with the A types, the 6:2 reassortants (HA and NA from recently circulating strains and the remaining 6 genes from A/PR/8/34 virus) are sometimes not easily obtained. This time-consuming process of reassortment is then followed by repeated passaging of the strain in embryonated eggs to allow for egg adaptation and growth enhancement. Influenza B virus prototype strains with good growth characteristics are usually obtained by direct and repeated passaging in embryonated eggs without attempting to generate reassortants. Although the manufacturing process is time-consuming, these killed influenza A and B virus vaccines are the workhorses for vaccination against influenza and have been shown time and again to be highly effective.

The second major class of viral vaccines consists of live viruses. The only FDA-licensed product against influenza is the cold-adapted attenuated vaccine. It is based on work originally done by Maassab's laboratory (*5*) and later by Murphy and colleagues (*6*). Influenza virus was passaged at 25°C in tissue culture (chicken kidney cells) and in embryonated eggs. This modified Jennerian approach resulted in a cold-adapted, temperature-sensitive, and highly attenuated master strain. The annually updated vaccine strains are generated in the laboratory by reassortment with viruses more closely related to the currently circulating ones. The resulting vaccine strains (both A and B types) are 6:2 reassortants with the 6 nonsurface protein genes derived from the cold-adapted master strains and the HA and NA from circulating A and B viruses, reflecting the changing antigenicity. These cold-adapted influenza virus vaccines are easily administered by nasal spray. They induce local mucosal neutralizing immunity and cell-mediated responses that may be longer lasting and more cross-protective than those elicited by chemically inactivated (killed) vaccine preparations. Vaccine efficacy in vaccine-naive children 6 months to 18 years of age is high (range 73%–96%). In children revaccinated for a second season, vaccine efficacy climbs to 82% to 100% (*7*).

## Need for Improvement?

Despite the obvious efficacy of both killed and live influenza virus vaccines, there is room for new developments. Among the critical issues in developing new and better vaccines are the following: price per dose, speed of production, ease of production, choice of substrates to grow the virus in or to express viral antigens, cross-protection for variant strains, efficacy in general and in immunologically naive populations, safety, and acceptance by the regulatory agencies and the public.

### New Adjuvants

Most of the current inactivated influenza virus vaccines do not contain an adjuvant. To stretch the available supply, antigen-sparing adjuvant approaches should be considered (*8*). Alum is an adjuvant that has been approved by the FDA for use in several vaccines. MF59, a proprietary adjuvant from Chiron (Emeryville, CA, USA), has also been successfully used in several countries (other than the United States). If, under adjuvant conditions, a fifth or a tenth of the antigenic mass currently present per vaccine dose (45 μg of HA protein) would suffice to stimulate an adequate protective response, a big supply problem would be solved.

Many adjuvants are now under investigation. Liposome-like preparations containing cholesterol and viral particles (immune-stimulating complexes) have been successfully used in mice (*9*) by subcutaneous and intranasal administration. Another adjuvant strategy involves the use of heat-labile *Escherichia coli* toxin complexed with lecithin vesicles and killed trivalent influenza virus preparations for intranasal administration (*10*). Although this specific vaccine has been withdrawn because of Bell's palsy cases associated with its administration, similar approaches may become more acceptable in the future if these safety issues can be resolved. Much work is also currently being conducted on synthetic adjuvants, such as synthetic lipid A, muramyl peptide derivatives, and cationic molecules (*11*). Also, Ichinohe et al. showed that poly (I:C) is a promising new and effective intranasal adjuvant for influenza virus vaccines (*12*).

### Genetically Engineered Live and Killed Influenza Virus Vaccines

As indicated, current FDA-licensed influenza vaccines are based on technologies developed in the 1960s and earlier. Through the breakthrough of reverse genetics techniques (*13**–**15*), infectious influenza viruses from plasmid DNAs transfected into tissue culture cells can now be rescued. This technology permits the construction of high-yield 6:2 seed viruses by mixing the 6 plasmid DNAs from a good-growing laboratory strain with the HA and NA DNAs obtained by cloning relevant genes from currently circulating viruses. Thus, within a 1- to 2-week period, the appropriate seed viruses could be generated for distribution to the manufacturers. The backbones of the 6:2 recombinant viruses could be prepared, tested, and distributed in advance. Similar approaches can be envisioned for the manufacturing of live, cold-adapted influenza virus vaccines. In this case, the backbone would consist of the 6 genes of the cold-adapted master strain. Again, the HA and NA of the currently circulating strains would be cloned and used for rescue in the plasmid-only reverse genetics system. Such an approach would have several advantages over the present manufacturing process. First, it would dramatically accelerate the timeframe for obtaining seed viruses for annual production and thus allow more time to select the appropriate antigenic seed strains. Second, it would standardize the seed viruses to be used. Regulatory agencies do not insist on a sequenced product to be given to humans but instead allow only partially characterized products for annual immunization. Third, DNA cloning may eliminate any adventitious agents present in the throat washings of the original isolate. Finally, in the case of the current highly pathogenic H5 strains, viruses with that HA (containing a multibasic HA1/HA2 cleavage site) kill embryonated eggs, making it difficult to use eggs as growth substrate. Also personnel involved in manufacturing those vaccines might be in danger of becoming infected. Thus, the HA of these virulent strains will need to be modified. Removal of the basic cleavage peptide by reverse genetics results in a virus that is attenuated for embryonated eggs, thus allowing high yields to be attained. Modification by reverse genetics results in a product that is easier to manufacture and safer to handle (this includes safety considerations for all persons working with the virus).

## Live Influenza Virus Vaccines with Altered Nonstructural Protein 1 Genes

The ability to site specifically engineering changes in the influenza virus genome also allows us to consider novel vaccine approaches. We have demonstrated that the nonstructural protein 1 (NS1) of influenza viruses has interferon antagonist activity (*16*). Influenza viruses that lack NS1 cannot counter the interferon response of the host. Thus, infection of cells with a virus that lacks NS1 results in the induction of interferon and blockage of virus replication. When truncations are made in NS1, viruses are generated with an intermediate activity, which enables them to replicate in the host and also to induce an interferon response. By engineering a virus with intermediate virulence and ability to induce interferon, one can construct ideal influenza virus vaccines that are both attenuated and highly immunogenic (*17**–**20*). Interferon appears to be an excellent adjuvant that enhances production of immunoglobulins and contributes to the activation of dendritic cells required for antigen presentation (*21**–**23*). We thus believe that, per virus particle made or antigen molecule delivered, the immune response will be enhanced compared to that of conventional live or killed virus vaccines. This process should translate into lower doses of live virus vaccine required to induce a robust and protective immune response. If a hundredfold lower dose is required, many more people could have access to influenza virus vaccines. This issue is clearly of paramount importance in the event of a new pandemic virus. Moreover, a live virus vaccine may give protective immunity in immunologically naive populations after a single administration, while killed virus vaccines may require high antigenic doses and a prime-boost regimen to protect against a pandemic strain. It may turn out that only live influenza virus vaccines can provide the necessary protection in case of a new pandemic. Because live influenza virus vaccines appear to be more effective in immunologically naive populations and they can be intranasally administered, they would represent a more economical way of vaccinating large numbers of people.

### Replication-defective Vaccines

Other promising approaches concern the use of replication-deficient preparations. For example, virus particles that lack the gene for the nuclear export protein (NEP; formerly NS2) will go through a single cycle of replication (without forming infectious particles) (*24*). Virus particles without the M2 gene may also fit this formula (*25*). Mass production of defective viruses can be achieved by using complementing cell lines. The administration of virosomes (consisting of reconstituted viral envelopes that lack RNA), and the use of viruslike particles made by expression of viral proteins have also been shown to be effective immunization strategies against influenza (*26**,**27*). Yet another approach concerns DNA vaccination in humans by using plasmids that express >1 foreign gene. Unfortunately, this approach has been less than convincing since it appears to work best in mice and other small mammals (*28*). Thus, the jury is still out as to whether this approach is reasonable for improving influenza virus vaccines in humans.

## Universal Vaccines?

Influenza viruses continue to undergo antigenic drift, which is mostly reflected in accumulating changes in the HA. This fact requires us to change the vaccine formulation or at least to reexamine the seed strains on an annual basis. Unfortunately, predicting the evolutionary change of the viral HA has not been reliable (*29*). Thus, short of developing 20/20 foresight, predicting strain variation or the emergence of a particular pandemic strain (avian or otherwise) is unlikely (*30*). A more realistic approach is the design of more cross-protective vaccines for use in interpandemic years and during pandemics. Neirynck et al. have designed vaccines based on the conserved extracellular portion of the M2 protein fused to the hepatitis B core protein (*31*). Such an immunogen may induce a cross-reactive response in the vaccinated host. Similarly, immunization with the NA antigen is likely to induce responses that are more cross-reactive than those by the more variable HA (*32*). In both cases, however, protection will require immune responses that are more vigorous than what is seen after natural infection. Antibodies against NA and M2 proteins in infected humans are generally not protective. Thus vaccines consisting of NA or M antigens would need to be made to induce a dramatically enhanced immune response. Alternatively, genetically engineered viruses could be generated, which would express several variant antigens or epitopes, thereby achieving a more cross-protective immunization. Chimeric HA recombinant viruses that express an additional 140 amino acids have recently been described (*33*). Such genetically engineered viruses may present several conserved immunogenic epitopes on the viral surface, which would be a first step toward a more universal influenza vaccine.

## Conclusions

Technologies are now in place to design and construct new influenza virus vaccines that have the potential to be cheaper and more cross-protective than current vaccine preparations, while at the same time being equally safe. The greatest problems for new and better vaccines appear to be associated with regulatory hurdles and the lack of an adequate market. Regarding the bureaucratic restrictions levied on vaccines by licensing agencies, the message has to come through "that small risks have to be tolerated for larger ones to be avoided" (*34*). Also, the message needs to be disseminated to the general public that vaccines have the best cost-benefit ratio of any medical treatment and that limitations of the tort law should be considered where vaccines are concerned. The public often views vaccines and prophylactic treatments in general as being of low priority. Many people also believe they should be free. Thus, the absence of a robust commercial market is a major difficulty, resulting in slow progress for research and development of new influenza vaccines and in dangerously thin supply lines. In fact, we are far from being prepared to deal with regular influenza outbreaks, and adequate measures to cope with a pandemic outbreak are only now being considered, but are not yet in place (35,36; and http://www.washingtonpost.com/wp-dyn/content/article/2005/11/01/AR2005110101100.html).
